# Do Animals Engage Greater Social Attention in Autism? An Eye Tracking Analysis

**DOI:** 10.3389/fpsyg.2020.00727

**Published:** 2020-06-16

**Authors:** Georgitta J. Valiyamattam, Harish Katti, Vinay K. Chaganti, Marguerite E. O’Haire, Virender Sachdeva

**Affiliations:** ^1^Department of Applied Psychology, Gitam University, Visakhapatnam, India; ^2^Centre for Neuroscience, Indian Institute of Science, Bengaluru, India; ^3^Department of Commerce, Osmania University, Hyderabad, India; ^4^Department of Comparative Pathobiology, Purdue University College of Veterinary Medicine, West Lafayette, IN, United States; ^5^Child Sight Institute, Nimmagadda Prasad Children’s Eye Care Centre, L V Prasad Eye Institute, GMRV Campus, Visakhapatnam, India

**Keywords:** animals, autism (ASD), social attention, visual attention, eye tracking, human animal interaction (HAI), neurobiomarker

## Abstract

**Background:**

Visual atypicalities in autism spectrum disorder (ASD) are a well documented phenomenon, beginning as early as 2–6 months of age and manifesting in a significantly decreased attention to the eyes, direct gaze and socially salient information. Early emerging neurobiological deficits in perceiving social stimuli as rewarding or its active avoidance due to the anxiety it entails have been widely purported as potential reasons for this atypicality. Parallel research evidence also points to the significant benefits of animal presence for reducing social anxiety and enhancing social interaction in children with autism. While atypicality in social attention in ASD has been widely substantiated, whether this atypicality persists equally across species types or is confined to humans has not been a key focus of research insofar.

**Methods:**

We attempted a comprehensive examination of the differences in visual attention to static images of human and animal faces (40 images; 20 human faces and 20 animal faces) among children with ASD using an eye tracking paradigm. 44 children (ASD *n* = 21; TD *n* = 23) participated in the study (10,362 valid observations) across five regions of interest (left eye, right eye, eye region, face and screen).

**Results:**

Results obtained revealed significantly greater social attention across human and animal stimuli in typical controls when compared to children with ASD. However in children with ASD, a significantly greater attention allocation was seen to animal faces and eye region and lesser attention to the animal mouth when compared to human faces, indicative of a clear attentional preference to socially salient regions of animal stimuli. The positive attentional bias toward animals was also seen in terms of a significantly greater visual attention to direct gaze in animal images.

**Conclusion:**

Our results suggest the possibility that atypicalities in social attention in ASD may not be uniform across species. It adds to the current neural and biomarker evidence base of the potentially greater social reward processing and lesser social anxiety underlying animal stimuli as compared to human stimuli in children with ASD.

## Introduction

Autism Spectrum Disorder (ASD) is a pervasive developmental disorder emerging in the first three years of life and characterized by significant deficits in social functioning and repetitive patterns of interest and behavior (American Psychiatric Association, 2013). Atypical visual attention has been widely identified as a key behavioral hallmark in the autism diagnosis (Jones et al., 2008). From infancy, typical individuals show a preference for social stimuli, including faces (Haith et al., 1977; Brosch et al., 2007), face-like configurations (Farroni et al., 2005; Johnson, 2005; Frank et al., 2009, 2014), and biological motion (Simion et al., 2008). Further, typical individuals also show a greater visual attention to the eyes, with a greater preference for direct gaze, thus demonstrating a greater sensitivity to eye contact and a greater capacity to decode information derived through it (Farroni et al., 2002; Dubey et al., 2015). Evidence suggests that these social preferences may be phylogenetically preserved (Emery, 2000; Rosa Salva et al., 2011), enabling the systematic specialization of social brain networks that orient infants toward social signals (Decasper and Fifer, 1980; Klin et al., 2002, 2009; Simion et al., 2008).

Children with ASD on the contrary, show atypical visual processing patterns as early as from 2 to 6 months of age (Jones and Klin, 2013), with differences becoming clearly discernible toward the end of the first year of life (Osterling and Dawson, 1994; Osterling et al., 2002). Studies employing static (e.g., Dalton et al., 2005), dynamic (e.g., Klin et al., 2003) and interactive stimuli (e.g., Campbell et al., 2014) have evidenced a significantly decreased attention to the eyes and direct gaze in children with ASD when compared to typical controls. This was also accompanied by an increased attention to typically less salient parts of the face including the nose, mouth and other facial regions along with an increased attention to the body and other objects (Joseph and Tanaka, 2002; Klin et al., 2002; Dalton et al., 2005). The visual atypicalities in individuals with ASD form part of an overall reduced social inclination as seen in deficits in pointing, showing objects, looking at others, orienting to name, social smiling, shared affect and social vocalizations (Dawson et al., 1998; Zwaigenbaum et al., 2005; Ozonoff et al., 2010; Wan et al., 2013). Early emerging neurobiological deficits in perceiving social stimuli and direct gaze as rewarding (e.g., Osterling et al., 2002; Scott-Van Zeeland et al., 2010; Kohls et al., 2013), or an active avoidance of direct gaze due to the potential threat perceived and the anxiety it entails (e.g., Kliemann et al., 2010; Tanaka and Sung, 2013; Tottenham et al., 2013), have been widely purported as potential reasons for the atypicality in visual gaze patterns in individuals with ASD. While a majority of studies point to atypical visual attention, a few studies also report near normal attentional preferences in individuals with ASD, both in terms of overall preferences to social stimuli as also the preferences to salient regions within a social stimulus (e.g., Bar-Haim et al., 2006; Fletcher-Watson et al., 2009). However studies also report that an employment of more sensitive measures reveals subtle differences in social attention in ASD, which may have important implications (e.g., Fletcher-Watson et al., 2009).

While the initial evidence base for atypical visual processing in ASD has emerged from retrospective studies of home video tapes (e.g., Osterling and Dawson, 1994; Zwaigenbaum et al., 2005), more recent studies using eye tracking measures have also substantiated these findings that were initially obtained through observational and naturalistic paradigms (Papagiannopoulou et al., 2014; Chita-Tegmark, 2016). For instance Chawarska and Shic (2009) using an eye tracking paradigm on 2- and 4-year-old children with ASD, not only revealed atypical attention patterns in young children, but also an intensification of the symptoms with age, with a lesser attention to core internal features of the face, like the eyes and a greater attention to external features such as hair, cheeks and other aspects of the image such as the body and screen (Chawarska and Shic, 2009). While the atypicality in social attention in ASD has been well-documented, whether this atypicality persists equally across species types or is confined to humans has not been a key focus of research insofar.

The benefit that animal companionship can provide to human wellbeing is a field of growing enquiry and the focus of the multidisciplinary field of human animal interaction (HAI). The HAI theory suggests that humans seek out contact with animals as they represent a source of social support that is calming, accepting and patient (Norris et al., 1999; Kruger and Serpell, 2010). In fact a key benefit that companion animals provide across population groups is their capacity to enhance social functioning. Animals can act as social facilitators, motivating positive social participation and reducing social withdrawal which can be crucial for vulnerable population groups such as those with disabilities, particularly when the possibilities for human social support are uncertain and/or deficient (Eddy et al., 1988).

Research evidence reports similar robust social functioning benefits of animal companionship for children with ASD, with the presence of animals leading to increased social skills, positive affect, and positive social behaviors along with lower levels of negative affect and social anxiety (Bass et al., 2009; Prothmann et al., 2009; Beetz et al., 2012; O’Haire et al., 2013; Funahashi et al., 2014). For instance, in a study involving social interaction situations between ASD and TD (typically developing) children, unique anxiolytic effects with a 43% decrease in skin conductance responses was observed in children with ASD when guinea pigs were present compared to toys, indicative of significantly lesser social anxiety and consequently enhanced social functioning (O’Haire et al., 2015). A key hormone implicated in these positive effects is oxytocin, with studies pointing to the potential of interactions with animals in increasing oxytocin levels in humans (Odendaal, 2000; Odendaal and Meintjes, 2003; Gordon et al., 2011; Handlin et al., 2011). This can have important implications considering that oxytocin pathways are critically implicated in social reward processing deficits in ASD (Modi and Young, 2012) and hence also atypical visual attention (Kanat et al., 2017). The social benefits that animals provide may also emerge from the fundamental morphological and behavioral characteristics that animals possess which can effectively engage with the low arousal levels that characterize children with ASD. For instance, animals such as dogs represent a multisensory stimulus, have simpler movements that are easier to interpret and may possess a higher level of behavioral and structural neoteny emerging from selective breeding (Redefer and Goodman, 1989; Rogers, 1998; Solomon, 2010; Silva et al., 2011) and these factors may also operate for several other domesticated animal species. The biophilia hypothesis (Wilson, 1984) proposes another interesting possibility of enhanced attention in the presence of animals, which can be crucial to social interactions. It suggests that humans are genetically predisposed with an innate tendency to focus on life and life-like processes (Wilson, 1984; Kellert and Wilson, 1993) due to the evolutionary benefits it provided in terms of procuring food and ensuring species survival. Several research studies also support this possibility and report that the presence of an animal leads to a heightened social awareness in children with ASD (Martin and Farnum, 2002).

Research evidence of the greater social benefits that animals provide becomes extremely significant as social impairments and accompanying social isolation are key deficit areas that characterize the ASD diagnosis (e.g., Carter et al., 2005; American Psychiatric Association, 2013). The capacity for animals to elicit greater social motivation and lesser social anxiety also points to the interesting possibility of a greater social reward value or a lesser gaze aversion for animal stimuli thereby potentially evoking a lesser level of atypicality in its visual processing.

A recent study by Whyte et al. (2016) examined this possibility providing neurobiological evidence that children with ASD may perceive greater social reward from animal faces, compared to human faces, as indicated by greater activation in the amygdala and putamen. Additional evidence on the same lines has emerged from eye tracking studies. A study comparing face scanning in children with ASD, ADHD (attention deficit hyperactivity disorder) and typical controls, revealed that while ASD children showed atypical patterns of face processing, they also looked at dog images the most, as compared to human faces. This preference for animal images was also shared by TD children and those with ADHD in the study (Muszkat et al., 2015). Another study comparing children with ASD and TD children, revealed among other findings that ASD children looked significantly longer at the eyes than other face and body parts in animal pictures, whereas no particular area of interest was significantly focused on in human pictures (Grandgeorge et al., 2016). The results thus indicate differences in visual gaze exploration by children with ASD depending on whether a picture represented a human or an animal face, and a potentially lesser atypicality in the viewing of animal faces by children with ASD as compared to when they viewed human faces.

Against this backdrop, the present study attempts a comprehensive examination of the differences in visual attention to human and animal stimuli among children with ASD, overcoming limitations of existing studies in terms of a lack of consistent stimulus dimensions and characteristics across human and animal images (Muszkat et al., 2015; Grandgeorge et al., 2016) and an absence of the examination of gaze patterns to direct and averted gaze images. Based on the well-documented phenomenon of atypical visual attention in ASD and existing evidence of the greater social inclination toward animals in children with ASD, our primary hypothesis is that while typical children will show greater overall social attention to all images when compared to children with ASD, the latter will show a greater visual preference for animal images when compared to human images.

## Method

### Ethics

All protocols to be adopted in the study, including the procedures for obtaining informed consent were approved by the Institutional Ethics Committee of Andhra University and the Ethical Review Board of LV Prasad Eye Institute, Visakhapatnam. Informed consent was obtained in writing from the principals of three special schools and one regular school participating in the study. Informed consent was also obtained in writing from the parents/caregivers for the inclusion of the individual participants along with verbal assent from the participants. To ensure a complete understanding of the protocols involved, informed consent forms were provided in both English and Telugu (local language) languages where required.

### Participants

#### Recruitment and Eligibility

Participants in the study were recruited from three special education schools and one regular school in the city of Visakhapatnam, India. Inclusion criteria for participants with ASD included: (a) age between 5 and 12 years, (b) parent and/or teacher reported diagnosis of ASD, and (c) normal or corrected to normal vision (essential for valid observations on the eye tracker), as examined by a certified optometrist. Inclusion criteria during data analysis included (a) a score of ≥11 on the Social Communication Questionnaire (SCQ) and ≥70 on the Social Responsiveness Scale (SRS-2) (detailed descriptions of the SCQ and the SRS-2 provided in section Screening Measures), to indicate a diagnosis of ASD. Exclusion criteria for participants with ASD included (a) a co-morbid diagnosis of congenital deafness, mental retardation, seizure disorder and any acute medical, genetic conditions or psychiatric conditions such as schizophrenia, (b) an inability to follow instructions, (c) visual issues amounting to a lack of normal or corrected to normal visual capacity, and (d) an inability to achieve calibration on the eye tracker.

Inclusion criteria for typically developing (TD) participants included (a) age between 5 and 12 years, (b) no parent and/or teacher reported diagnosis of ASD, and (c) normal or corrected to normal vision as examined by a certified optometrist. Inclusion criteria during data analysis included a score of ≤10 on the Social Communication Questionnaire (SCQ) and ≤69 on the Social Responsiveness Scale (SRS-2), to indicate the absence of an ASD diagnosis and social deficits.

#### Sample Characteristics

A total of 54 children with ASD and 47 typical children participated in the study. During the process of data collection 33 children with ASD were excluded from the sample for the following reasons: (a) 2 did not meet the criteria for ASD diagnosis on the SCQ and the SRS-2, (b) 26 participants with ASD did not meet the criteria for normal or corrected to normal vision due to previously undiagnosed refractive errors and strabismus, (c) 3 participants were unable to follow instructions during the process of eye tracking, and (d) 2 could not achieve calibration on the eye tracker. Similarly, 24 typical children were excluded from the sample as they did not meet the criteria for normal or corrected to normal vision due to previously undiagnosed refractive errors. The final sample of participants with ASD consisted of 21 children (*M* = 16, *F* = 5) between 6 and 12 years of age (mean age 10.03 years). In addition to the existing diagnosis (psychiatrists, clinical psychologists, pediatrician reports), all 21 participants met the criteria for a diagnosis of ASD on the SCQ and the SRS-2. The final sample of typical participants consisted of 23 children (*M* = 9, *F* = 14) between 6 and 12 years of age (mean age- 9.34 years) and without a diagnosis of ASD in terms of either prior parent and/or teacher reports or scores on the SCQ and SRS-2. Table 1 summarizes the demographic, IQ and ASD screening data for the participants in this study.

**TABLE 1:** Demographic details of the participants.

**Characteristic**	**ASD (*n* = 21)**	**TD (*n* = 23)**	***t-*value (*p*-value)**
Age	10.03 (1.60)	9.34 (1.52)	1.47
Gender	16M/5F	9M/14F	–
**Social communication questionnaire (SCQ)**			
Total	18.66 (4.04)	4.04 (1.29)	16.46**
**Social responsiveness scale (SRS)**			
*T-*score (full-scale)	78.47 (4.56)	45.60 (4.73)	23.44**
**Raven’s colored progressive matrices (CPM)**			
Percentile description	ASD (*n* = 21)		TD (*n* = 23)
	*Between 10th–25th percentile (n* = *18) Grade IV*		*Between 25th and 50th percentiles (n* = *16) Grade III-*
	*At or below 10th percentile- Grade IV- (n* = *03)*		*Between 50th and 75th percentiles (n* = *07) Grade III* +
	*Below Average Intellectual Capacity*		*Intellectually Average*

*ASD, Autism Spectrum Disorder; TD, Typically Developing; n, Sample Size; Scores in the cells represent means and standard deviations unless otherwise noted.* *p ≤ 0.05, **p ≤ 0.01.

### Measures

#### Visual Gaze Fixation Measure

Participants’ visual gaze fixation patterns were captured using non-invasive Tobii X3-120 eye tracker and the Tobii Pro Studio Software (Tobii, Stockholm, Sweden). The Tobii X3-120 eye tracker uses infrared light emitting diodes (LEDs) to detect corneal reflection patterns and these along with other visual data are collected by the image sensors and processed to situate the participant’s gaze point on the screen at a sampling rate of 120 hz per second. The Tobii X3-120 provides highly accurate and precise data with a high freedom from head movement rate (19.7″× 15.7″− width × height), making it suitable for use with children with developmental disabilities (Tobii, Stockholm, Sweden) and has been widely used in research with children diagnosed with ASD and other developmental disabilities (e.g., Riby and Hancock, 2009; Sasson et al., 2011; Pierce et al., 2016).

#### Screening Measures

Two standardized measures were used for the purpose of autism screening. These include:

##### The Social Communication questionnaire (SCQ).

The SCQ is a brief and widely used ASD screening tool based on the Autism Diagnostic Interview-Revised (Lord et al., 1994). It is a 40 item questionnaire to be completed by the parent or caregiver. Each item is answered as “yes/no” and is scored either as 0 or 1 with 1 endorsing the presence of the autism symptom (Rutter et al., 2003). The SCQ is an extremely well validated screening instrument (Norris and Lecavalier, 2010) and has a high agreement with ADI-R scores (Le Couteur et al., 2003). It shows excellent autism screening properties in discriminating between ASD and non-ASD cases with a reported sensitivity and specificity of 0.85 and 0.75 (Berument et al., 1999) and 0.88 and 0.72 (sensitivity and specificity) respectively (Chandler et al., 2007). While the current form of the SCQ asks for the presence of behaviors in the past three months, the lifetime version assesses presence of symptoms across the child’s entire developmental history (Marvin et al., 2017) and hence is more suitable for screening. The SCQ can be used with children above 4 years of chronological age, on condition that their mental age is above 2 years (Rutter et al., 2003). The lifetime version of the SCQ was used in the current study. Scores on the SCQ- Lifetime version range from 0 and 33 for non- verbal and 0–39 for verbal children. A cut-off score of ≥11 was used for the screening of ASD (Norris and Lecavalier, 2010).

##### The Social Responsiveness Scale (SRS).

The Social Responsiveness Scale (SRS-2) (Constantino and Gruber, 2012), formerly known as the Social Reciprocity Scale is a 65- item rating scale completed by a parent, teacher or other adult informant that uses a continuum approach to assess the severity of ASD symptoms in children aged 2.5–18 years. The SRS-2 provides a continuous measure of the severity of the child’s social impairments on a 4-point Likert scale scored from 0 (never true) to 3 (almost always true), yielding an overall severity score with a higher score corresponding to greater impairment. Scores on the SRS-2 can range from 0 to 195 with a higher score indicating greater impairment and corresponding T scores representative of the levels of autism severity. The subscales of the SRS-2 assess five domains namely, social awareness, social motivation, social communication and interaction, social cognition and autistic preoccupations as seen in restricted and repetitive behaviors (RRB) with the social communication and interaction subscale and the RRB subscale corresponding to the two main symptom domains of the DSM-5 (Constantino and Gruber, 2012; American Psychiatric Association, 2013). The SRS is a highly valid and quantitative measure and in addition to its use as a clinical diagnostic tool and a screening measure of autism severity (Constantino and Todd, 2005; Duvall et al., 2007; Frazier et al., 2014), it can also be used for assessing intervention efficacy, in terms of the response to intervention (Pine et al., 2006; Constantino et al., 2009). The SRS-2 has a high reliability (internal consistency reliability alpha 0.95 for all gender and age ranges) and inter-rater agreement validity coefficients ranging from 0.72 to 0.82. The present study used a cut-off score of ≥70 for ASD screening purposes (Constantino and Gruber, 2005).

#### IQ Assessment

##### Raven’s Colored Progressive Matrices (RCPM).

Raven’s Colored Progressive Matrices (RCPM) is a non-verbal test of intelligence and measures general cognitive and clear-thinking ability. The 36 items are arranged in three sets -A, Ab, and B consisting of 12 items each, and assess the chief cognitive processes of which children between 4 and 11 years of age, are usually capable. It can also be used with populations beyond this age range including adolescents and adults with mental or physical impairments and the elderly (Raven et al., 1996). The Raven’s CPM items seek to gauge cognitive development up to the point when an individual is satisfactorily and consistently able to engage in reasoning by analogy. The CPM produces a single raw score which can be converted to a percentile (Raven et al., 1996).

### Visual Stimuli

Stimuli presented to the participants consisted of static color photographs of humans and animals against a constant gray backdrop (29.5 × 32.5 degrees of visual angle). A total of 40 images were used in the study (humans = 20 images, males = 10, females = 10; animals total = 20 images, dogs = 8 cats = 8, horses = 2, and cows = 2) divided into an equal number of front facing and averted facing images (averted to the participant’s right). The human consisted of adult Indian male and female faces, obtained by the principal investigator with informed consent of the individuals who were photographed, whereas the animal images were procured from internet sources. All images were edited using Adobe Photoshop 7.0 to replace the background with uniform gray color (code#B6B5B5).

### Data Capture Procedures

**FIGURE 1:**
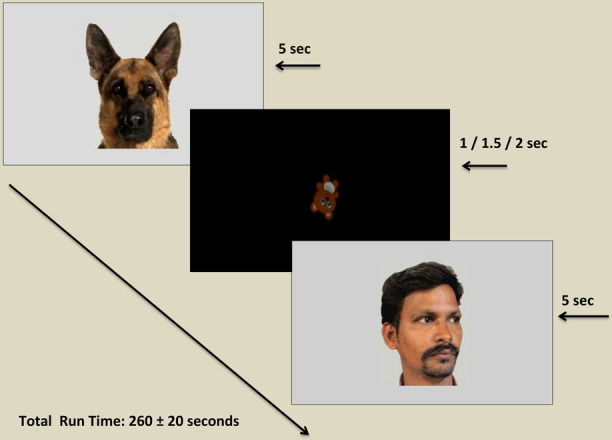
Diagram illustrating stimulus presentation within the eye tracking paradigm. Each target stimulus was displayed for a period of 5 s (5000 ms) followed by an inter-stimulus image displayed for a variable period of 1, 1.5, or 2 s. Permissions and image licenses have been obtained from the copyright holders (Source: ©Eric Isselee/Shutterstock.com).

The static human and animal images were presented to the participants on a 21.5″ high definition LCD monitor (with screen resolution 1920 × 1080 pixel) using Tobii Pro studio software. Participants were seated on a height adjustable chair either individually or in the lap of a caregiver or a research assistant, at an approximate distance of 60 cm from the screen. After a comfortable seating position was achieved, a manual five-point infant calibration was used for each participant wherein each child was instructed to follow an animated stimulus around the screen. In case of poor calibration, recalibration was conducted and the study was continued only for those participants who achieved a successful calibration as verified by the Tobii X3-120. After successful calibration, each target image was presented for 5 s and separated by a screen showing a tumbling teddy bear at the center of the screen, jittered at 1, 1.5, or 2 s, so as to re-center attention and to reduce the effect of possible distracters. The order of presentation of the stimuli was randomized across participants to counterbalance possible sequence effects. The total run time of the experiment was 260 ± 20 s (see Figure 1). The testing procedures for all participants were conducted in the same setting, free from distracters (e.g., noise and movement) and with optimal and constant illumination. Two research assistants were present in the room during the eye tracking procedure. One research assistant controlled the computer with the help of an extended screen and was seated behind a curtained panel invisible to the participant, while the other research assistant handled other logistics pertaining to the eye tracking process (e.g., handling the required seating adjustments for the participants and caregivers, seating participants in the lap if needed) without interfering with the procedure.

### Data Analysis

Regions of interest (ROIs) were drawn using interfaces provided by Tobii Studio©. ROI boxes encompassed the face including human hairline as well as ear or nose tips as applicable. Images were resized using Adobe Photoshop 7.0 so that ROI boxes were as near as possible to 600 × 850 pixels which would then correspond to 29.5 × 32.5 degrees of visual angle. Prior to testing the primary hypotheses, *post hoc* raw data export was done using Tobii Studio© software (Tobii, Stockholm, Sweden). The data export also included a fixation classification step that detected fixations based on the velocity of directional shifts of the eye (I-VT algorithm implemented in Tobii Studio). Custom scripts written in MATLAB© were used to extract and tabulate fixation related statistics. ROI-wise fixation statistics were tabulated in custom data structures as were dwell statistics obtained by collating fixations at different locations within an ROI, over a single presentation of an image. Fixations that did not land on face or face-part ROIs were assigned to a control “Screen” ROI. Image presentations, for which no fixation was made in any ROI, were not used for further analysis.

An initial examination of the data involving a computation of the Anderson- Darling test of normalcy and the Levene’s test for homogeneity of variances revealed a non-normal distribution with heterogeneous variances (*p* ≤ 0.05). To obtain a strong measure of the effects on the outcome variable while accounting for the data characteristics, the study used a regression analysis using the unbiased recursive binary partitioning approach (Hothorn et al., 2006) as a model for testing the *a priori* hypotheses. The recursive binary partitioning approach (BRP) and resulting tree models provide a robust and easily interpretable measure of all possible interaction effects across the variables of interest on the outcome measures with the flexibility of fewer assumptions regarding the data and evidence growing application and promising utility in psychological research (King and Resick, 2014). Unbiased binary recursive partitioning models use the algorithmic process of recursive partitioning in a binary fashion to create homogenous clusters of the data based on the outcome variables, in ways that maximize the differences between the clusters. The stepwise splitting process involves (a) a search for all possible splits across all variables, (b) identification of the most optimal split based on some criterion, (c) splitting the sample at this limit resulting in two daughter nodes, and (d) repeating steps 1–3 on the resulting daughter/intermediary nodes till terminal nodes are reached and no further partitioning is possible based on the predefined termination criteria (Martin, 2015). Unbiased binary recursive partitioning models are most suited for conditions where all possible interactions of independent variables for the data as a whole become the focus of interest, when the data satisfies fewer assumptions such as that of normality and homoscedasticity and the variables are measured categorically (Doove et al., 2014), as is the case in the present study.

**FIGURE 2:**
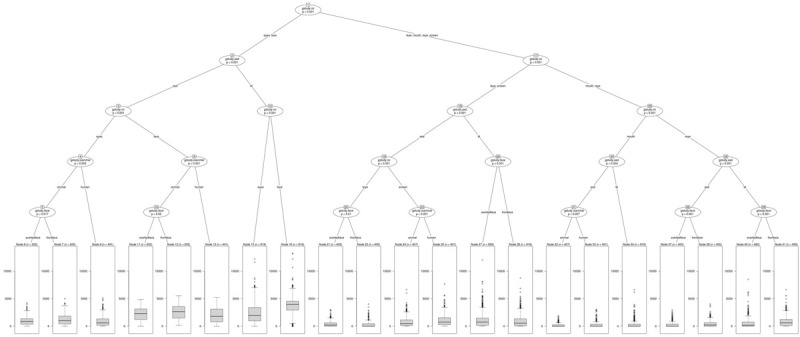
Conditional Inference Tree showing statistically significant interactions of the independent variables.

The present study attempted an analysis of all existing interaction effects of the independent variables –ROI (face, left eye, right eye, eye region, mouth and screen), stimulus type (human, animal), stimulus orientation (front facing, averted facing) and diagnosis (TD, ASD) on the dependent variable of fixation duration using the BRP approach. Fixation duration was defined with respect to the dwell timings falling within each ROI and data analysis focused on the fixation time in milliseconds for each independent observation event for every participant at the ROI level within each image presented, comprising a total number of 10362 valid observations. An observation was defined as the total dwell time within an ROI of an image shown to the participant. Figure 2 shows the conditional inference tree derived from this data. The most statistically significant split generated, partitioned ROIs as the root nodal point with a further binary branching down of the nodes by largest differences across possible groupings, till no more splits were possible with respect to the termination criteria, which was specified *a priori* as a p value exceeding 0.05. Data was analyzed using the R 3.4.3, Partykit version 3.2-2 which is a highly functional and flexible toolkit for summarizing, displaying and modifying recursive partitions and their tree based depictions (Hothorn and Zeileis, 2015).

## Results

Results obtained were examined at the level of the primary hypotheses (see Figure 2). Tables 2, 3 provide Mean and SD values for gaze fixation durations.

**TABLE 2:** Mean and SD values of gaze fixation duration on all ROIs of human and animal images for ASD participants.

**Diagnosis**	**ROI**	**Stimulus type**	**Gaze orientation**	**Mean (*SD*)**
ASD	Eye region	Animal	Avertedface	1011.86 (845.21)
			Frontface	1236.89 (1028.88)
		Animal total		1125.20 (947.77)
		Human	Avertedface	847.03 (904.63)
			Frontface	980.98 (992.99)
		Human total		913.84 (950.91)
	Eye region total			1020.31 (954.61)
	Face	Animal	Avertedface	2212.90 (1216.87)
			Frontface	2502.66 (1281.16)
		Animal total		2358.85 (1256.53)
		Human	Avertedface	1817.06 (1287.55)
			Frontface	2073.32 (1334.02)
		Human total		1944.87 (1315.57)
	Face total			2153.40 (1301.95)
	Left eye	Animal	Avertedface	471.88 (531.16)
			Frontface	366.67 (586.41)
		Animal total		418.89 (561.46)
		Human	Avertedface	385.46 (560.10)
			Frontface	271.4 (521.27)
		Human total		328.57 (543.41)
	Left eye total			374.06 (554.08)
	Mouth	Animal	Avertedface	156.38 (280.39)
			Frontface	190.44 (332.30)
		Animal total		173.54 (307.73)
		Human	Avertedface	217.31 (445.73)
			Frontface	296.71 (518.72)
		Human total		256.91 (484.54)
	Mouth total			214.92 (407.13)
	Right eye	Animal	Avertedface	264.63 (427.53)
			Frontface	500.07 (640.60)
		Animal total		383.22 (557.30)
		Human	Avertedface	280.36 (509.28)
			Frontface	402.15 (577.36)
		Human total		341.10 (547.03)
	Right eye total			362.32 (552.29)
	Screen	Animal	Avertedface	866.25 (996.28)
			Frontface	784.91 (932.29)
		Animal total		825.28 (964.25)
		Human	Avertedface	1142.81 (1220.25)
			Frontface	1010.44 (983.73)
		Human total		1076.79 (1109.21)
	Screen total			950.10 (1045.67)

**ASD, Autism Spectrum Disorder; ROI, Region of Interest; SD, Standard deviation (Total no. of Observations: 10362).**

**TABLE 3:** Mean and SD values of gaze fixation duration on all ROIs of human and animal images for TD participants.

**Diagnosis**	**ROI**	**Stimulus type**	**Gaze orientation**	**Mean (*SD*)**
ASD	Eye region	Animal	Avertedface	2296.53 (1819.72)
			Frontface	2244.86 (1484.61)
		Animal total		2270.75 (1659.39)
		Human	Avertedface	2263.83 (1717.42)
			Frontface	2417.99 (1632.88)
		Human total		2340.91 (1675.63)
	Eye region total			2305.87 (1667.00)
	Face	Animal	Avertedface	3807.43 (1531.11)
			Frontface	3854.70 (1116.14)
		Animal total		3831.02 (1338.98)
		Human	Avertedface	3468.86 (1539.48)
			Frontface	3830.02 (1460.41)
		Human total		3649.44 (1509.69)
	Face total			3740.13 (1429.10)
	Left eye	Animal	Avertedface	1153.04 (1292.08)
			Frontface	956.00 (988.63)
		Animal total		1054.73 (1153.70)
		Human	Avertedface	1195.35 (1310.15)
			Frontface	930.27 (1085.48)
		Human total		1062.82 (1209.06)
	Left eye total			1058.78 (1181.10)
	Mouth	Animal	Avertedface	291.84 (688.37)
			Frontface	285.16 (578.69)
		Animal total		288.51 (635.33)
		Human	Avertedface	241.41 (428.39)
			Frontface	365.37 (631.57)
		Human total		303.39 (542.60)
	Mouth total			295.96 (590.46)
	Right eye	Animal	Avertedface	606.44 (1011.74)
			Frontface	791.24 (808.78)
		Animal total		698.64 (919.78)
		Human	Avertedface	566.32 (987.46)
			Frontface	908.72 (1049.00)
		Human total		737.52 (1031.92)
	Right eye total			718.10 (977.18)
	Screen	Animal	Avertedface	909.09 (1078.73)
			Frontface	761.98 (1036.67)
		Animal total		835.69 (1059.36)
		Human	Avertedface	1114.32 (1388.32)
			Frontface	912.37 (1066.46)
		Human total		1013.35 (1240.67)
	Screen total			924.62 (1156.47)

**TD, Typically developing; ROI, Region of Interest; SD, Standard deviation (Total no. of Observations: 10362).**

Results indicated a significant interaction effect of diagnosis and ROI with typical children showing significantly greater visual attention to the face and eye region (node 2), left eye (node 18), right eye (node 35) (all *p* ≤ 0.001), and mouth (node 30, *p* ≤ 0.01) ROIs of all social images (human and animal images combined) as compared to children with ASD. Children with ASD showed a greater visual attention to the screen ROI (node 18, *p* ≤ 0.001) in all social images when compared to typical controls. No significant differences in visual attention were observed within typical children to any of the ROIs of human images when compared to animal images. However, within children with ASD, findings revealed a significant interaction between ROI and stimulus type, with ASD children showing a significantly greater visual attention to the face (node 9, *p* ≤ 0.001) and eye region (node 4, *p* ≤ 0.01) ROIs in animal images when compared to human images. Also, a significantly greater visual attention was seen to the mouth (node 30, *p* ≤ 0.01) and screen (node 18, *p* ≤ 0.001) ROIs in human images when compared to animal images in children with ASD.

**FIGURE 3:**
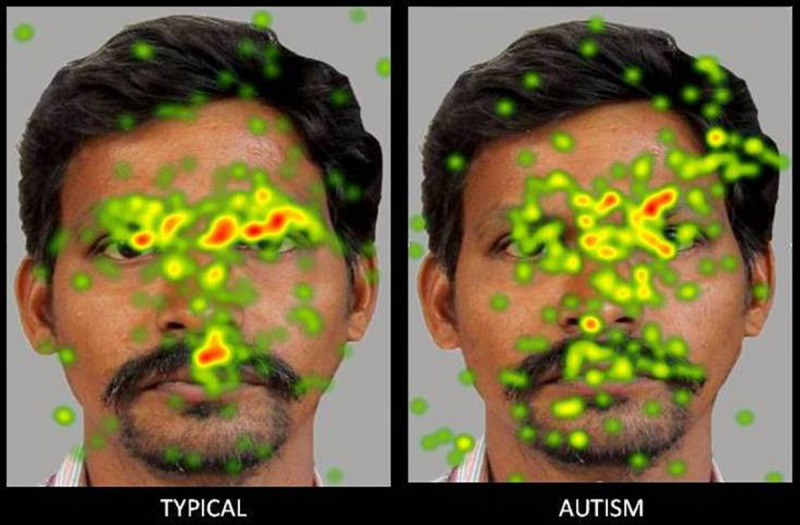
Heat Diagram illustrating the most attended areas of the human face by Typical and ASD participants and the lesser attention to salient regions of the human face in ASD. *(Gradients of most attended areas on the heat maps range from red through yellow to green).*

**FIGURE 4:**
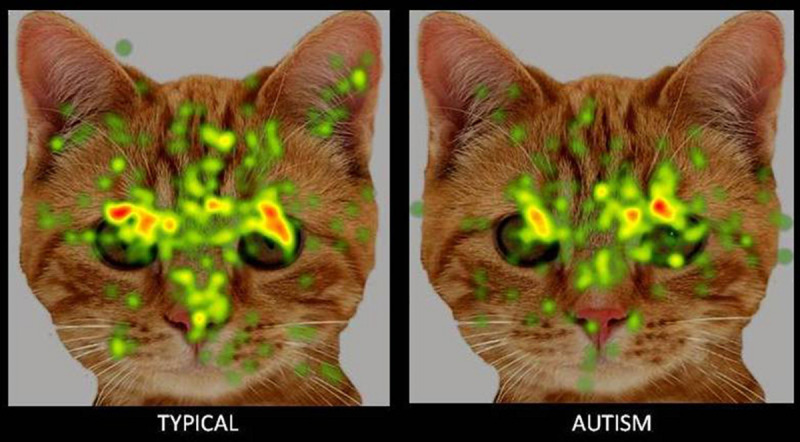
Heat Diagram illustrating the most attended areas of the animal face by Typical and ASD participants and the lesser attention to salient regions of the animal face in ASD. *Gradients of most attended areas on the heat maps range from red through yellow to green).*

**FIGURE 5:**
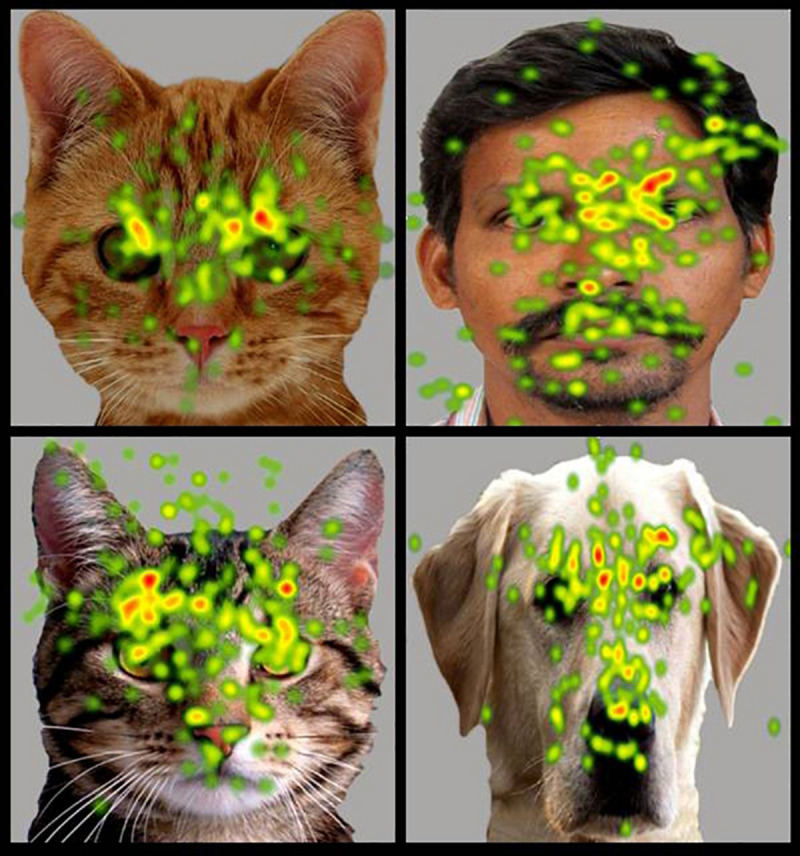
Heat Diagram illustrating the most attended areas of the human and animal faces by ASD participants and indicating the greater visual attention to salient regions of the animal face in ASD. (*Gradients of most attended areas on the heat maps range from red through yellow to green).*

**FIGURE 6:**
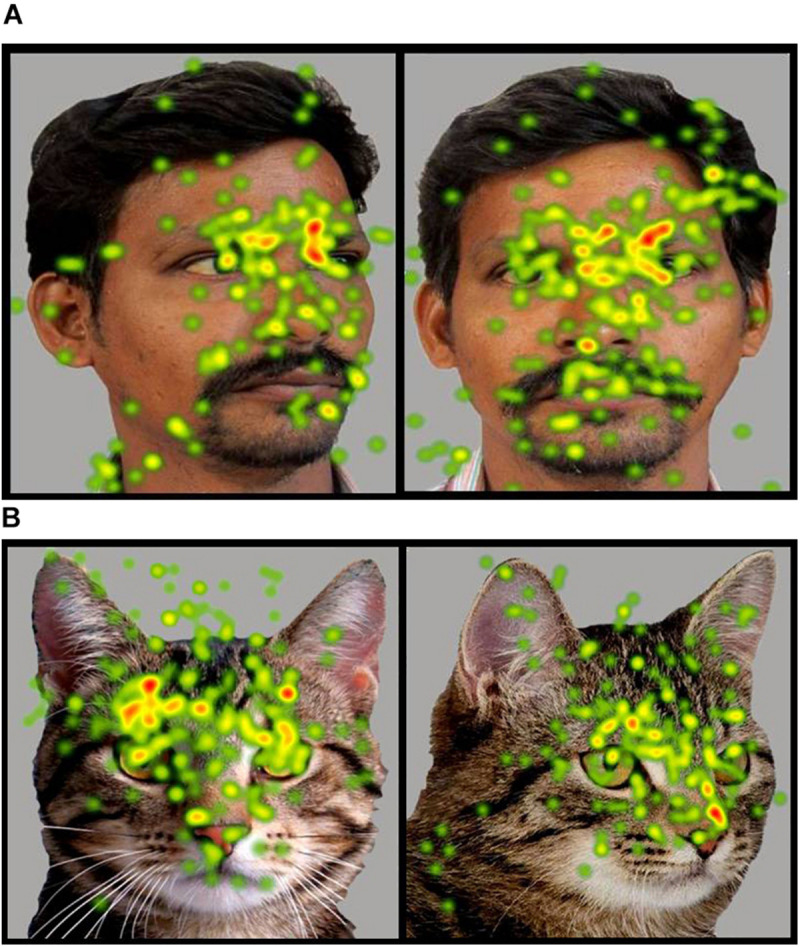
**(A,B)** Heat Diagrams illustrating the most attended areas of the human and animal front and averted faces by ASD participants and indicating the greater visual attention to direct gaze in the animal face in ASD *(Gradients of most attended areas on the heat maps range from red through yellow to green). Permissions and image licenses have been obtained from the copyright holders (Source: ©Ermolaev Alexander/Shutterstock.com).*

With regard to stimulus orientation, a significant interaction between ROI and stimulus orientation was observed within TD children with significantly greater visual fixation duration to the left eye and screen ROIs in averted social images (human and animals combined) when compared to front facing social images (node 26, *p* ≤ 0.001) and greater fixation to the right eye ROI in front facing social images (node 39, *p* ≤ 0.001). Similarly children with ASD also showed a greater visual fixation to the left eye ROI in averted facing images (node 20, *p* ≤ 0.01) and a greater visual fixation to the right eye ROI in front facing images (node 36, *p* ≤ 0.001). A significant interaction between ROI, stimulus type and stimulus orientation was observed within ASD children with a significantly greater visual attention allocation to the eye region (node 5, *p* ≤ 0.05) and face (node 10, *p* ≤ 0.05) ROIs of animal front facing images when compared to animal averted facing images. No significant differences were observed within TD and ASD groups for the mouth ROI on front and averted facing human and animal images. Figures 3–6B show heatmap images of the significant findings obtained.

## Discussion

The present study attempted a comparative examination of visual attention to animal and human faces in children with ASD and typical controls through an examination of six ROIs namely- face, left eye, right eye, eye region, mouth and screen. Results obtained reported a greater visual attention to faces in typical children when compared to children with ASD. Typical children showed significantly greater attention to the face, left eye, right eye and eye region ROIs and a lesser visual attention to the screen ROI indicative of a greater attention to the salient aspects of the images. Children with ASD on the contrary allocated comparatively greater attention than typical peers to the screen or the part of the image that did not contain socially significant information across all the images.

The results obtained are consistent with the bulk of previous eye tracking literature reporting atypical and lesser visual attention to social stimuli in children with ASD when compared to typical controls (Papagiannopoulou et al., 2014; Chita-Tegmark, 2016) and can be explained in terms of the marked deficits that children with ASD exhibit from an early age in preferentially orienting to social information and stimuli that are socially salient such as the face and the eyes (Klin et al., 2002; Jones and Klin, 2013; Campbell et al., 2014). Mixed results have been reported with regard to the mouth ROI with meta-analytic studies reporting greater, (Asberg Johnels et al., 2014; Grossman et al., 2015), lesser (Sterling et al., 2008; Rice et al., 2012) or no significant difference in attention allocation to the mouth in children with ASD when compared to typical controls (Wagner et al., 2013; Tenenbaum et al., 2014; Auyeung et al., 2015). Results in the present study revealed a significantly lesser visual fixation to the mouth in children with ASD when compared to typical children. Similar results have been found in earlier studies. For instance, Rice et al. (2012) found that in response to naturalistic social stimuli in a free viewing eye tracking paradigm, children with ASD showed a lesser attention to the mouth along with lesser attention to other regions of the face such as the eyes (Rice et al., 2012). Studies also suggest that the reduced attention allocation to the face in ASD is generalized, may not be specific to the eyes and may impact all salient areas of the face including the mouth, with the mouth not acting as a compensatory source of social information (Chita-Tegmark, 2016).

A key aim of the present study was to examine possible biases in social attention to animal faces in children with ASD when compared to human faces. Results obtained revealed a significantly greater attention allocation to animal faces and eye region and lesser attention to the animal mouth in children with ASD when compared to human faces, indicative of a clear attentional preference to socially salient regions of animal stimuli. These results are in line with previous research reporting a greater preference and more positive appraisal of animal stimuli over human and inanimate stimuli in children with autism and developmental disabilities. For instance preference studies in experimental settings using either images (Celani, 2002) or live stimuli (Prothmann et al., 2009) have reported an enhanced preference for animals in children with ASD over inanimate objects and human stimuli. Supportive research evidence has also emerged in the form of enhanced social functioning and reduced social stress in the presence of animals in children with ASD as revealed in both observational paradigms and through an examination of biomarker indices including salivary cortisol and skin conductance (O’Haire, 2013, 2017). A third line of supportive evidence has most recently emerged from brain imaging paradigms with sMRI and fMRI recordings reporting a greater activation in social reward and emotional arousal-specific areas of the brain in response to animal but not human stimuli (Whyte et al., 2016). Studies using eye tracking methods have also reported a greater visual attention to animal faces over human faces in children with ASD (Muszkat et al., 2015; Grandgeorge et al., 2016). The present study sought to substantiate these findings through a comprehensive eye tracking examination of visual gaze patterns to human and animal stimuli. It also aimed to extend these findings through an examination of gaze orientation in addition to stimulus type.

The positive attentional bias toward animal images was also seen in gaze orientation, in terms of a significantly greater visual attention to the face and eye region of front facing animal images when compared to animal images with averted eyes. However, no such preference for a direct gaze orientation was seen for human faces in children with ASD. Earlier research has reported that while typical children show an advantage with direct gaze and a disadvantage with averted gaze in visual tasks, this is not evident in children with ASD (Senju et al., 2003, 2005). Further children with ASD also showed gaze aversion effects in terms of an exaggerated stress response to direct gaze (Kylliäinen and Hietanen, 2006). Findings in the present study also showed no significant preference among children with ASD for direct gaze in human faces. However a preferential attention to direct gaze emerged for animal faces indicating the possibility of a significantly lesser amount of gaze aversion and social stress or a greater social reward in the presence of animal stimuli.

The greater attention to direct gaze in animals and a greater attention to the eye region in animal faces when compared to human faces can be cumulatively suggestive of that fact that children with ASD have a greater capacity to derive information from the eyes in animal stimuli – a capacity that is significantly impaired in the case of human stimuli. Verbal reports obtained from the participants and caregivers prior to the start of the experiment revealed that while none of the participants had pets, all of them had a history of positive interactions with the animal species whose images comprised the visual stimuli in the eye tracking procedure. There were no reports of aversive incidents with animals experienced by any of the participants. Considering that all children had a positive familiarity toward the animal species whose images were used as visual stimuli, the increased attention toward the salient parts of the animal images cannot be better explained in terms of curiosity or fear experienced when viewing the animal image. While typically developing children in the present study did not show a significantly greater attention to front facing stimuli across all the images, the significantly lesser attention to the screen in front facing images may be indicative of a greater focus on more salient locations of the face within the image, as compared to the relatively non-significant portions of the screen.

Possible explanatory paradigms for the enhanced interest in animals in children with ASD primarily include the biophilia hypothesis and the possible roles of oxytocin and neoteny. The biophilia hypothesis refers to the instinctive urge that we are hardwired with, to connect and affiliate with nature and other life forms including animals (Wilson, 1984). Another powerful explanation for the enhanced social interest to animal stimuli in typical individuals emerges from research evidence of the enhanced activation of key oxytocin and glutamatergic pathways in the presence of animals (Odendaal and Meintjes, 2003; Beetz et al., 2012). Increase in the secretion of these key neuropeptides has a direct modulatory effect on the enhancement of social reward perception, social bonding, reduced social anxiety, increased social behaviors and eye contact (Uvnäs-Moberg et al., 2000; Kosfeld et al., 2005; Uvnas-Moberg and Petersson, 2005). Because of its role in so many aspects of social functioning, researchers have considered the oxytocinergic system to be a principal point of treatment for disorders involving atypical social behavior and functioning (Fineberg and Ross, 2017). The fact that children with ASD share similar social benefits in the presence of animals as do typical individuals, places the possibility of an enhanced activation in these pathways as a potential explanation for the enhanced visual attention to animals in children with ASD. Research with typical individuals including children has also assigned neoteny a partial role in the human attraction and affinity toward animals. Neoteny refers to the presence of structural and/or behavioral infantile features in animals into adulthood due to their conscious or unconscious selective preference during domestication and breeding (Belyaev, 1979; Frank and Frank, 1982; Archer and Monton, 2011; Beck, 2014). Considering that children with ASD often show similar positive responses to animals as typical children, the impact of neoteny can be seen as a possible explanatory factor in the preferential visual attention toward animal stimuli in this study. However it should also be considered as a limited explanatory factor as not all animal images used in the study had high levels of morphological/structural neoteny (e.g., cows and horses) although the element of behavioral neoteny may have played a role since all children had a limited yet positive familiarity with the animal species whose images were incorporated in the visual stimuli.

Earlier eye tracking literature, though limited, has reported a comparatively increased visual attention to animal images when compared to human images in typical children (Muszkat et al., 2015; Grandgeorge et al., 2016), explained through the possible impact of biophilia and neoteny. However findings in the present study did not report any similar significant increases in the attention allocation to animal faces in typical children. However an examination at the trend levels indicated a relatively greater attention allocation to the face and lesser attention allocation to the screen in animal stimuli when compared to human stimuli indicative of partially higher, though non-significant attentional preference to animal faces in typical children.

Across social stimuli, both children with ASD and typical controls showed a significantly greater visual attention to the left eye in averted images and the right eye in front facing images. The bias to the left eye (from the side of viewer and the right eye of the image) can be explained in terms of the significantly greater exposure that the left eye received as compared to the right eye in averted images due to the image orientation, hence leading to greater fixation durations. However, an interesting finding is the significantly enhanced visual attention to the right eye (the right eye from the viewer’s perspective and the left eye of the image) in all front facing social images in both children with ASD and typical controls. Several research studies have reported the existence of a left gaze bias in both humans and other species including rhesus monkeys and dogs (Guo et al., 2009). The left gaze bias indicates a quicker and longer direction of attention to the right side of the person’s face toward whom attention is focused which also comprises the left side or the left hemifield from the perspective of the viewer (Grega et al., 1988; Burt and Perrett, 1997; Philips and David, 1997; Butler et al., 2005; Butler and Harvey, 2006). It is often considered the natural outcome of hemispheric lateralization of face perception functions (Guo et al., 2012). However, an opposite phenomenon was observed in the present study and would merit further investigation in subsequent research.

The study revealed a small number of outliers to the total gaze time of 5 s or 5000 ms. Several reasons may have led to this effect. During image presentation in the Tobii software, transition effects are observed in the close of an object and the start of the interstimulus interval, which here consisted of a movie with a centered tumbling teddy bear. Fixation events were counted if they started when the social stimulus was still present although some part of it may have extended into the interstimulus interval duration and resulted in outliers.

Also, unintentional technological inconsistencies may operate, thereby increasing the chances of error, such as the possibility of undetected background applications, or the computer being able to detect an eye tracker whereas Tobii studio being unable to (Tobii Error Codes, (n.d); Tobii Pro X3-120 Eye Tracker User Manual, (n.d)). These errors in technology, beyond the control of the investigator may also have contributed to the outlier effects. The outliers were, however, very few when compared to the number of valid observations obtained on the experiment.

While this study reveals differences in visual gaze patterns to animal and human stimuli in children with ASD and offers possible explanations for this phenomenon, it is limited in its capacity to identify the exact factors within the stimuli or the viewer that may underlie this difference. However, the principal aim of the present study was to examine possible differences in eye gaze patterns and based on the findings obtained herein, future studies can attempt a replication of these findings to assess its reliability across contexts and focus on possible causative elements that may trigger these differences. Although visual atypicalities in autism are evident as early as 6–12 months of age and clearly distinguishable by 12 months of age, (e.g., De Giacomo and Fombonne, 1998; Chawarska and Volkmar, 2005; Werner and Dawson, 2005; Zwaigenbaum et al., 2005), we focused primarily on participants in the middle and late childhood period. The present study was part of a larger project that also looked at the efficacy of animal-assisted intervention (AAI) for children with autism and considering the AAI component to be involved, selection of this age group was more appropriate. It would be interesting to examine if the patterns of gaze allocation seen in this study also hold for children at an earlier developmental level. The sample of children with ASD in this study also consisted of those with moderate-to-severe autism and whether the preferential attention to animals as seen in this study is also evident across differing levels of autism severity, merits investigation. Further while the children in both the groups were matched in terms of chronological age, the mental age of children with ASD was lower than their typical counterparts. Recent research suggests that lower mental age and intellectual disability may be more common than estimated in children with autism and that lower mental age with ASD may form a more severe diagnostic criteria that merits more specific intervention (e.g., Hinnebusch et al., 2017; Thurm et al., 2019). In another line of research, studies involving individual with autism with normal to high intelligence have revealed social functioning benefits including lesser stress in the presence of an animal (e.g., Wijker et al., 2019). The enhanced gaze allocation to animal stimuli even if emerging from a lower mental age may then signify patterns that may be widely shared among children with autism with important implications for intervention. Future research can also examine the implications of heterogeneity in mental age as a modulating factor in visual attention to animals in children with autism.

While the number of males was higher among children with ASD which is also reflective of the clinical presentation of ASD in a general population (Fombonne, 2009), the group of typical controls consisted of a higher number of females. Research on sex differences in visual attention to social stimuli with typical populations including children and infants have put forward mixed results with some studies reporting heightened attention to faces in females (Lutchmaya et al., 2002; Gluckman and Johnson, 2013; Harrop et al., 2019) whereas other studies have put forward contrary findings (Escudero et al., 2013). Recent research involving children with ASD has reported a comparatively greater attention to social stimuli in females as compared to males (e.g., Harrop et al., 2019). The sex differences in the composition of the two groups in the present study may have contributed to the between group differences in visual attention across social stimuli in children with ASD and typical controls. However the prime focus of the present study was to investigate whether differences exist in the visual attention to human and animal social stimuli and a fundamental finding is of the existence of these differences within children with ASD. It would be interesting for subsequent research to expand on the findings obtained herein so as to include an examination of the possible sex differences that may also exist. It would also be interesting to examine the possibilities of the enhanced visual attention and attraction to animals resulting in potential social facilitation effects.

## Conclusion

Overall the present study suggests that animal faces may elicit greater visual attention in children with ASD. It also suggests the possibility that the deficits in social functioning and related deficits in visual attention that children with ASD experience may not be uniform across species. The study adds to the current neural and biomarker evidence base of the potentially greater social reward processing and lesser social anxiety underlying animal stimuli as compared to human stimuli. Social impairments being a key area of autistic symptomatology, and considering the detrimental role of atypical visual attention in triggering, fostering and consolidating social deficits in ASD individuals, the evidence of enhanced visual attention to animals can have important implications in the planning of early interventions for children with ASD. It can also provide a strong evidence base for the use of AAI for autism that focuses on incorporating animals in the treatment process for children with ASD. Incorporation of animals may lead to an enhanced visual attention and preference to the context and activities involved, and thereby potentially lead to an overall enhancement in social attention.

## Data Availability Statement

The datasets generated for this study are available on request to the corresponding author.

## Ethics Statement

The studies involving human participants were reviewed and approved by Andhra University Institutional Ethics Committee, LV Prasad Eye Institute (LVPEI) Institutional Ethics Committee. Written informed consent to participate in this study was provided by the participants’ legal guardian/next of kin. Written informed consent was obtained from the individual(s) for the publication of any potentially identifiable images or data included in this manuscript.

## Author Contributions

GV conceptualized and designed the project, conducted the review of relevant literature, carried out the experiments, participated in the analysis and interpretation of the data, and drafted the manuscript. HK participated in the design of the project, in the carrying out of experiments, in the analysis and interpretation of the eye tracking data, and in the writing of the manuscript. VC participated in the analysis and interpretation of the data, the collection of relevant literature and in the writing of the manuscript. MO’H participated in the conceptualization of the project and reviewed the manuscript. VS designed and conducted the ophthalmologic assessments and reviewed the manuscript. All authors contributed to and have approved the final manuscript.

## Conflict of Interest

The authors declare that the research was conducted in the absence of any commercial or financial relationships that could be construed as a potential conflict of interest.

## Abbreviations

ASD: Autism Spectrum Disorder
TD: Typically Developing
BRP: Binary Recursive Partitioning
ROI: Region of Interest
sMRI: Structural Magnetic Resonance Imaging
fMRI: Functional Magnetic Resonance Imaging.
