# Corrigendum: Do Animals Engage Greater Social Attention in Autism? An Eye Tracking Analysis

**DOI:** 10.3389/fpsyg.2020.584787

**Published:** 2020-09-16

**Authors:** Georgitta J. Valiyamattam, Harish Katti, Vinay K. Chaganti, Marguerite E. O'Haire, Virender Sachdeva

**Affiliations:** ^1^Department of Applied Psychology, Gitam University, Visakhapatnam, India; ^2^Centre for Neuroscience, Indian Institute of Science, Bengaluru, India; ^3^Department of Commerce, Osmania University, Hyderabad, India; ^4^Department of Comparative Pathobiology, Purdue University College of Veterinary Medicine, West Lafayette, IN, United States; ^5^Child Sight Institute, Nimmagadda Prasad Children's Eye Care Centre, L V Prasad Eye Institute, Visakhapatnam, India

**Keywords:** animals, autism (ASD), social attention, visual attention, eye tracking, human animal interaction (HAI), neurobiomarker

In the original article, there was an error. The visual angle was incorrectly reported as 13.92 × 19.35. It has now been corrected to 29.5 × 32.5.

A correction has been made to Methods (Section), Visual Stimuli (Sub-section), Paragraph no.1 and to Methods (Section), Data Analysis (Sub-section), Paragraph no. 1.

Paragraphs:

## Visual Stimuli

Stimuli presented to the participants consisted of static color photographs of humans and animals against a constant gray backdrop (29.5 × 32.5 degrees of visual angle). A total of 40 images were used in the study (humans = 20 images, males = 10, females = 10; animals total = 20 images, dogs = 8 cats = 8, horses = 2, and cows = 2) divided into an equal number of front facing and averted facing images (averted to the participant's right). The human consisted of adult Indian male and female faces, obtained by the principal investigator with informed consent of the individuals who were photographed, whereas the animal images were procured from internet sources. All images were edited using Adobe Photoshop 7.0 to replace the background with uniform gray color (code#B6B5B5).

## Data Analysis

Regions of interest (ROIs) were drawn using interfaces provided by Tobii Studio©. ROI boxes encompassed the face including human hairline as well as ear or nose tips as applicable. Images were resized using Adobe Photoshop 7.0 so that ROI boxes were as near as possible to 600 × 850 pixels which would then correspond to 29.5 × 32.5 degrees of visual angle. Prior to testing the primary hypotheses, *post hoc* raw data exportwas done using Tobii Studio© software (Tobii, Stockholm, Sweden). The data export also included a fixation classification step that detected fixations based on the velocity of directional shifts of the eye (I-VT algorithm implemented in Tobii Studio). Custom scripts written in MATLAB© were used to extract and tabulate fixation related statistics. ROI-wise fixation statistics were tabulated in custom data structures as were dwell statistics obtained by collating fixations at di?erent locations within an ROI, over a single presentation of an image. Fixations that did not land on face or face-part ROIs were assigned to a control “Screen” ROI. Image presentations, for which no fixation was made in any ROI, were not used for further analysis.

The authors apologize for this error and state that this does not change the scientific conclusions of the article in any way. The original article has been updated.

